# On the Mechanisms of the Cardiotoxic Effect of Lead Oxide Nanoparticles

**DOI:** 10.1007/s12012-023-09814-5

**Published:** 2023-12-18

**Authors:** Ilzira A. Minigaliyeva, Svetlana V. Klinova, Marina P. Sutunkova, Yuliya V. Ryabova, Irene E. Valamina, Ivan G. Shelomentsev, Tatiana N. Shtin, Tatiana V. Bushueva, Yuri L. Protsenko, Alexander A. Balakin, Ruslan V. Lisin, Daniil A. Kuznetsov, Boris A. Katsnelson, Liubov V. Toropova

**Affiliations:** 1 Yekaterinburg Medical Research Center for Prophylaxis and Health Protection in Industrial Workers, Yekaterinburg, Russian Federation 620014; 2 Laboratory of Stochastic Transport of Nanoparticles in Living Systems, Ural Federal University, Yekaterinburg, Russian Federation 620000; 3 Ural State Medical University, Yekaterinburg, Russian Federation 620109; 4 Institute of Immunology and Physiology, Ural Branch of the Russian Academy of Sciences, Yekaterinburg, Russian Federation 620049; 5 Laboratory of Mathematical Modeling of Physical and Chemical Processes in Multiphase Media, Ural Federal University, Yekaterinburg, Russian Federation 620000; 6 Otto-Schott-Institut für Materialforschung, Friedrich-Schiller-Universität-Jena, 07743 Jena, Germany

**Keywords:** Cardiotoxicity, Nanoparticles, Experimental study, Rats

## Abstract

Lead compounds are one of the most common pollutants of the workplace air and the environment. In the occupational setting, the sources of their emission, including in nanoscale form, are various technological processes associated with lead smelting and handling of non-ferrous metals and their alloys, the production of copper and batteries. Both lead poisoning and lead exposure without obvious signs of poisoning have a detrimental effect on the cardiovascular system. The purpose of this research was to investigate the mechanisms of the cardiotoxic effect of lead oxide nanoparticles (PbO NPs). The toxicological experiment involved male albino rats subchronically exposed to PbO NPs (49.6 ± 16.0 nm in size) instilled intraperitoneally in a suspension. We then assessed post-exposure hematological and biochemical parameters of blood and urine, histological and ultrastructural changes in cardiomyocytes, and non-invasively recorded electrocardiograms and blood pressure parameters in the rodents. Myocardial contractility was studied on isolated preparations of cardiac muscles. We established that PbO NPs induced oxidative stress and damage to the ultrastructure of cardiomyocytes, and decreased efficiency of the contractile function of the myocardium and blood pressure parameters. We also revealed such specific changes in the organism of the exposed rats as anemia, hypoxia, and hypocalcemia.

**Graphical Abstract:**

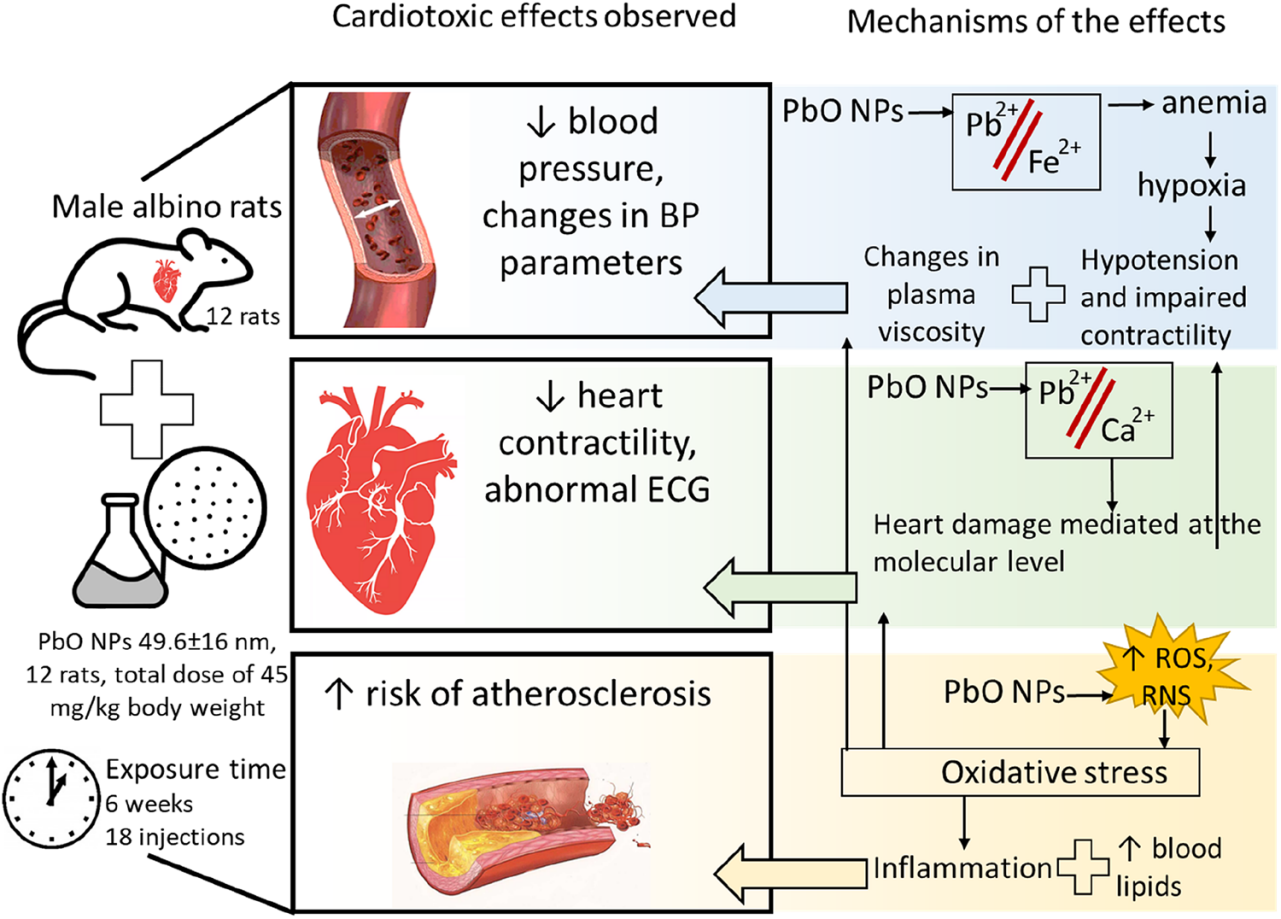

## Introduction

Lead (Pb) and its compounds, including in nanoscale form, are a well-known pollutant of the workplace air and the environment. Miller et al [1] in their study of exposures in a refinery found a high number of nanoparticles in workplace aerosols, the latter being rich in metals including silver, lead, antimony, selenium, and zinc.

Published data indicate that nanoparticles (NPs) of a specific metal and its oxide form have a much more pronounced harmful effect on target species including humans than corresponding microparticles [2–6]. Metal oxide NPs tend to exhibit a lower degree of stability and more susceptible to dissolution and release of ions in biological fluids (than metal NPs comprised of a single metal element), which leads to the formation of reactive oxygen species (ROS) and oxidative stress in cells [7]. Along with the effects common for all Me NPs, specific ones, related to the identity of the forming metal in any chemical form, have been revealed [8–10]. The latter necessitates consideration of a cardiovasotoxic effect of lead, not limited to its nanosized form.

The toxicity of lead and its compounds has been reported in numerous *in vitro* and *in vivo* studies. Interactions with DNA [11, 12] and oxidative stress [11] are among the adverse health effects of this metal. Lead exposure has been shown to induce inaccurate replication and transcription of genes, while Pb ions disrupt the balance of the antioxidant system [12]. The influence of lead on the cardiovascular system has received little attention in recent publications. Yet, a causal relationship has been demonstrated between human exposure to lead and the prevalence of arterial hypertension [13–17]. An *in vivo* study of male albino rats exposed to PbO NPs by oral gavage for four weeks showed an increase in blood pressure parameters and oxidative stress in heart tissues, and the development of cardiac muscle hypertrophy [18].

Despite the attention of researchers to the impact of occupational and environmental hazards on cardiac dysfunction, the mechanisms of its development have been poorly studied so far. According to the results of a few in vivo studies, possible mechanisms of lead-induced hypertension include the triggering of oxidative stress, impaired calcium regulation, inflammation, dysfunction of the vascular endothelium with a decrease in the bioavailability of nitric oxide and impaired signalling cascades with its participation, an increase in adrenergic activity, and a change in renin-angiotensin system [19–26]. The mechanisms of the effect of lead on the heart muscle have been studied even less. The data obtained by different researchers are contradictory. Fioresi et al [27] found changes in calcium (Ca^2+^) kinetics in cardiomyocytes in the absence of an inotropic toxic effect, while Silva et al [28] observed an adverse inotropic effect of lead associated with impaired contractile mechanism in cardiomyocytes in similar studies. Thus, the purpose of this study was to identify the mechanisms of the cardiotoxic effect of PbO NPs.

## Materials and Methods

### Method of Preparation and Physicochemical Characteristics of Lead Oxide (PbO) Nanoparticles

The suspension of lead oxide nanoparticles was prepared by laser ablation of thin metal sheet targets of 99.99 % pure lead in sterile deionized water using the technique described elsewhere [29].

The shape and size of the particles were established by scanning electron microscopy.

The stability of the suspensions was characterized by zeta potential measured by a Zetasizer Nano ZS analyzer (Malvern, UK).

### Experimental Animals and Toxicity Modeling

The experiments were carried out on outbred male rats aged 3 months with the initial body weight of 222.31 ± 4.68 g, 12 individuals per group.

The rats were kept in a separate room of the vivarium of our Center; they breathed unfiltered air and were given bottled artesian water and standard balanced feed.

The experiments were designed and carried out in accordance with the International Guiding Principles for Biomedical Research Involving Animals developed by the Council for International Organization of Medical Sciences and the International Council for Laboratory Animal Science (2012) and approved by the Ethics Committee of the Yekaterinburg Medical Research Center for Prophylaxis and Health Protection in Industrial Workers (Protocol No. 2 of February 1, 2018).

Each animal received group and individual labels marking group affiliation and an individual number in the experiment. Suspensions of PbO nanoparticles (0.5 mg/mL) were instilled intraperitoneally thrice a week during six weeks (18 injections in total) at a single dose of 2.5 mg/kg body weight making up a total dose of 45 mg/kg body weight. A pilot had been previously conducted to select the dose. The experimental dose was determined as such causing adverse changes in a number of functional and biochemical parameters of the rodent but no severe poisoning with a fatal outcome. Repeated intraperitoneal injections in rats simulated a long-term regular exposure of industrial workers to PbO NPs. Six weeks of life of a mature rat corresponds to approximately four years of human life [30].

### Assessment of the Health Status of Experimental Animals

#### Non-Invasive Recording of ECG and Blood Pressure Parameters

At week 5 of the experiment, electrocardiogram (heart rate, voltage and time parameters (P wave and QRST complex), and changes in the isoelectric line) and blood pressure (systolic, diastolic, mean arterial pressure, blood flow velocity, tail blood volume, and heart rate) parameters in rats were registered non-invasively using the ecgTUNNEL (emka TECHNOLOGIES, Paris, France) and CODA-HT8 (Kent Scientific, Torrington, USA) instruments, respectively.

#### Determination of Blood and Urine Parameters in Rats

We measured blood levels of total protein, albumin, globulins, calcium, alanine and aspartate aminotransferases (ALT, AST), gamma-glutamine transferase (GGT), lactate dehydrogenase (LDH), catalase, ceruloplasmin, high-density lipoproteins (HDL), creatinine kinase, and creatinine kinase-MB, myoglobin, troponin I, natriuretic peptides, angiotensin-converting enzyme (ACE), vascular endothelial growth factor (VEGF), and endothelin-1 [31].

The Mythic 18 fully automated hematology analyzer was used to measure hemoglobin, hematocrit, mean erythrocyte volume, plateletcrit, platelets, leukocytes, and erythrocytes. Reticulocytes were counted routinely. A cytochemical determination of the activity of succinate dehydrogenase (SDH) in blood lymphocytes was carried out, based on the reduction of para-nitrotetrazolium violet to formazan and counting the granules of the latter during optical microscopy with immersion [32].

To measure blood lead levels, 1 mL of rat blood was diluted three times with concentrated nitric acid and 2 mL of concentrated hydrogen peroxide was added after 2 hours. After two more hours, the test portion was dried at temperatures up to 400°. The dry residue was then dissolved in 5 mL of distilled water. Blood lead levels were measured using the Agilent 7800 ICP-MS instrument (Agilent, Malaysia).

#### Coproporphyrin and δ-ALA

Urinary creatinine, coproporphyrin, and delta-aminolevulinic acid (δ-ALA) levels were measured following the exposure to PbO NPs [33].

#### Histological Evaluation

Heart tissues from four rats in each experimental group were prepared for microscopic histological examination by the hematoxylin and eosin stain. We used the Avtandilov’s planimetric ocular grid and the image recognition programmed system CellSens (Olympus, Hamburg, Germany) for morphometric characterization.

#### Isolated Multicellular Myocardium Preparations

**Fig. 1:**
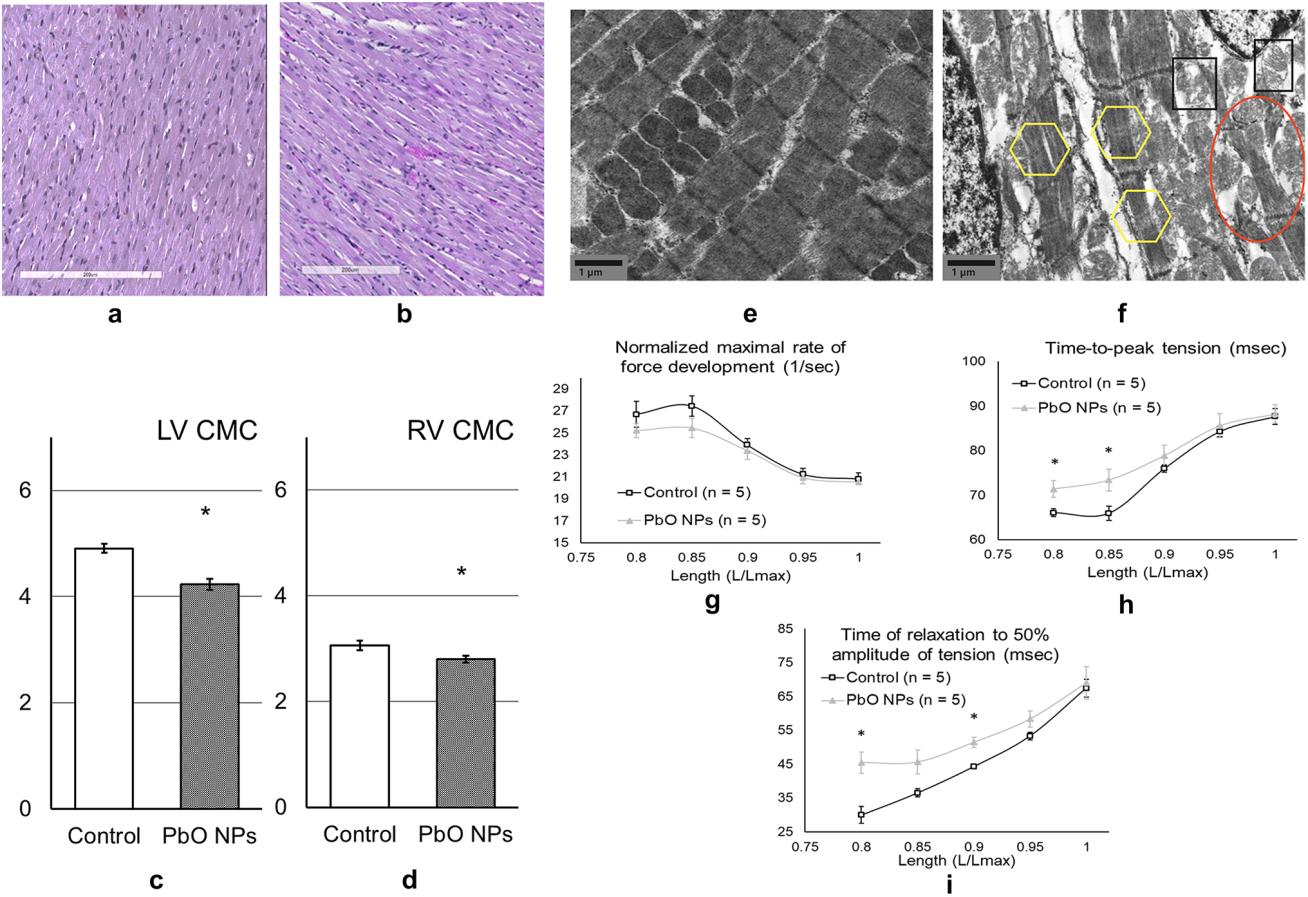
Myocardium of the left ventricle of (**a**) control and (**b**) PbO NP exposed rats. Hematoxylin and eosin stain; magnification *200; Thickness of cardiomyocytes of the left (**c**) and right (**d**) ventricles of rats; *p < 0.05 compared with the controls; STEM-изoбpaжeния кapдиoмиoцитoв (yвeличeниe *20 000) (**e**) in the control group, demonstrating well-defined Z-band, myofibrils in regular rows, and normal, dense-matrix mitochondria with uniform cristae filling; (**f**) in the experimental group of rats exposed to PbO NPs, showing a local loss of clarity of the outline of Z-bands (yellow figures), a slight loss of myofibrils while maintaining their normal location (orange figure), slight swelling of mitochondria with a light matrix and loss of inner space filling density (black figures); Velocity (**g** – maximum force velocity) and temporal (**h** – time-to-peak tension, **i** – time of relaxation to 50% of the maximum amplitude of tension) parameters of isometric contraction and relaxation of the papillary muscles of the right ventricle of the heart in rats of the control and PbO NP exposure groups (color figure online)

Myocardial contractility was assessed on isolated multicellular myocardium preparations [34]. Thin trabeculae and papillary muscles were dissected from the right ventricle of the same heart. The preparations were fixed to two rods of the length servomotor and force transducer in a thermo-controlled bath (Muscle Research System, Scientific Instruments GmbH, Germany). The experiments were performed in a modified Krebs–Henseleit solution (in mM: NaCl 118.5; NaHCO_3_ 14.5; KCl 4.2; KH_2_PO_4_ 1.2; MgSO_4_ 1.2; glucose 11.1, CaCl_2_ 1.9) oxygenated by a mixture of 95 % O_2_ and 5 % CO_2_, pH = 7.4 at 35 °C. All measurements were taken at the pacing frequency of 2 Hz, temperature of 35 °C, and the working length of 0.95 Lmax. The recording of isolated muscle contractions was carried out in isometric (Fig. 1a) and physiological modes (Fig.1b,c). The mechanical response under either isometric or physiological mode of contraction was measured using an analog-to-digital and digital-to-analog converter (PCI–1716S, AdLink Technology Inc., Taiwan) at a frequency of 10 kHz. The force was normalized to the estimated cross-sectional area to obtain mechanical tension values. To estimate force development and relaxation rates, the time course of isometric contraction was normalized to its amplitude.

In the physiological mode, a sequence of loads similar to the physiological sequence in the cardiac cycle was applied to the muscle, which allowed measuring force–velocity relationships (under different afterloads) and force-shortening loops, which resemble the pressure–volume loop typical of the whole heart [35].

#### Scanning Electron Microscopy

We conducted an electron microscopy study of post-exposure ultrastructural pathological changes in the heart as described elsewhere [29, 36].

#### Statistical Data Analysis

The Student’s t test was used to determine the significance of differences between the means in the exposure and control groups of rats with a p value of 0.05 or less considered statistically significant.

The data are presented as Mean ± Standard Error (SE).

## Results

### Nanoparticle Parameters

The mean diameter of the lead oxide nanoparticles used was 49.6 ± 16 nm. The nanoparticle shape was spherical. The stability of the suspensions was characterized as high by zeta potential up to 42 mV. The high stability allowed us to increase the concentration of the suspension to 0.5 mg/mL by its partial evaporation at 50 °C without changes in the size and chemical identity of the NPs.

### ECG and Blood Pressure Parameters

Table 1 shows electrocardiogram parameters of the experimental animals showing increasing trends in the QT interval, the amplitude of the T wave, and the duration of the QRS interval.

**Table 1:** Electrocardiogram (Lead II) parameters and hemodynamic parametrsin rats following the exposure to lead oxide nanoparticles (Mean ± SE)

Parameters	Control group	PbO NP exposure group
Rats in group, *n*	12	12
Electrocardiogram (Lead II) parameters		
Heart rate, beats per minute	411.34 ± 14.93	379.46 ± 11.01
Macruz index	0.58 ± 0.05	0.64 ± 0.04
RR, ms	147.48 ± 5.77	155.29 ± 3.12
P duration, ms	16.73 ± 0.74	17.39 ± 0.86
PQ, ms	46.05 ± 1.49	44.98 ± 1.72
**QRS**, ms	**23.86 ± 0.51**	**24.92 ± 0.24**
**QT**, ms	**67.22 ± 1.40**	**69.28 ± 1.91**
Corrected QT interval (Bazett formula)	176.02 ± 6.00	174.41 ± 6.64
Corrected QT interval (Fridericia formula)	127.67 ± 3.72	128.18 ± 4.40
Isoelectric line, mV	− 0.0693 ± 0.0055	− 0.0611 ± 0.0041
P amplitude, mV	0.0915 ± 0.0057	0.0788 ± 0.0030
R amplitude, mV	0.392 ± 0.027	0.371 ± 0.022
S amplitude, mV	0.009 ± 0.009	− 0.029 ± 0.021
QRS amplitude, mV	0.405 ± 0.025	0.341 ± 0.030
** T amplitude, mV**	**0.141 ± 0.025**	**0.149 ± 0.013**
Hemodynamic parameters		
Systolic blood pressure, mm Hg	154.96 ± 6.80	**128.48 ± 3.77**
Diastolic blood pressure, mm Hg	122.01 ± 3.01	**91.09 ± 4.08**
Mean blood pressure, mm Hg	134.71 ± 3.07	**103.22 ± 3.80**
Heart rate, beats per minute	317.85 ± 14.55	301.56 ± 11.77
Tail blood flow velocity, µL/min	25.38 ± 3.21	22.16 ± 1.65
Tail blood volume, µL	105.60 ± 13.19	78.81 ± 3.77

Bold values indicate statistical significance

* *p* < 0.05 based on Student’s t-test

After intraperitoneal instillation of lead oxide nanoparticles to rats, changes in some hemodynamic parameters were observed, 50 % of which were altered statistically compared with the control group (Table 1).

### Hematological Parameters

We observed an increase in the activity of such enzymes as creatinine kinase, lactate dehydrogenase, aspartate aminotransferase, and gamma-glutamyltranspeptidase in blood serum of the exposed rats (Table 2), while bioenergetic processes assessed by the activity of SDH in blood lymphocytes remained unchanged. The activity of catalase and ceruloplasmin in blood serum indicating the antioxidant status of the body demonstrated a decrease.

**Table 2:** Hematological parameters and levels of blood lead and serum Ca^2+^ in rats following subchronic exposure to lead oxide nanoparticles (Mean ± SE)

Parameters	Control group	PbO NP exposure group
Rats in group, *n*	12	12
Hematological parameters		
Hemoglobin, g/L	155.71 ± 6.93	**132.25 ± 2.69**
Serum myoglobin, ng/mL	210.42 ± 75.38	**366.00 ± 59.06**
Hematocrit, %	22.89 ± 1.24	**19.53 ± 0.42**
Reticulocytes, ‰	23.53 ± 2.42	**38.45 ± 3.12**
Platelets, 10^3^/µL	704.00 ± 28.67	**862.63 ± 37.95**
Plateletcrit, %	0.20 ± 0.01	**0.24 ± 0.01**
Monocytes, 10^9^/L	0.417 ± 0.030	**0.518 ± 0.034**
Serum albumin, g/L	48.48 ± 0.69	**43.18 ± 0.48**
Albumin/Globulin index	1.65 ± 0.06	**1.35 ± 0.06**
Succinate dehydrogenase activity, formazan granules per 50 lymphocytes	460.95 ± 15.04	466.89 ± 30.39
Serum AST, mM/h × L	200.67 ± 16.97	217.40 ± 16.84
Serum gamma-glutamyltransferase, U/L	0.25 ± 0.11	0.63 ± 0.26
Serum lactate dehydrogenase, U/L	2,760.67 ± 220.52	3,133.69 ± 272.27
Serum catalase, µmol/L	0.58 ± 0.01	**0.45 ± 0.05**
Serum ceruloplasmin, mg/%	166.19 ± 14.49	118.09 ± 26.85
Serum high density lipoproteins, mmol/L	1.28 ± 0.07	1.12 ± 0.07
Serum creatine kinase, U/L	2943.43 ± 653.67	3170.25 ± 746.33
Serum creatine kinase MB, U/L	1637.52 ± 204.52	1657.69 ± 185.28
Serum troponin I, ng/mL	0.14 ± 0.09	0.12 ± 0.05
Serum natriuretic peptides, pg/mL	1.50 ± 0.27	0.92 ± 0.12
Serum angiotensin-converting enzyme, U/L	210.06 ± 16.07	**159.54 ± 14.84**
Serum vascular endothelial growth factor, IU/mL	2.03 ± 0.43	2.50 ± 0.67
Plasma endothelin-1, pg/mL	46.20 ± 2.94	**19.06 ± 1.72**
Levels of blood lead and serum Ca^2+^		
Blood lead level, µg/L	1.80 ± 0.52	507.14 ± 38.18*
Serum Ca^2+^, mmol/L	2.61 ± 0.02	2.48 ± 0.05*

Bold values indicate statistical significance

* *p* < 0.05 based on Student’s t-test

We measured the blood lead level in the rats as a marker of lead exposure and found that it was statistically higher in the exposed rodents compared with the controls (Table 2). At the same time, we observed a decrease in the level of calcium in blood serum of the exposed animals.

### Coproporphyn and δ-ALA

We found an increase in the level of δ-aminolevulinic acid in urine of the exposed rats. The urinary level of coproporphyrin did not change compared with the control rodents (Table 3).

**Table 3:** Levels of urinary coproporphyrin and δ-aminolevulinic acid measured in the rats (Mean ± SE)

Parameters	Control group	PbO NP exposure group
Rats in group, *n*	12	12
Urinary coproporphyrin, nmol/L	93.25 ± 37.98	69.99 ± 9.09
δ-aminolevulinic acid in urine, µg/mL	8.62 ± 1.78	**78.13 ± 14.33**

Bold value indicates statistical significance

* *p* < 0.05 based on Student’s *t*-test

### Histological Evaluation and Scanning Electron Microscopy

We found signs of atrophy on histological sections of the myocardium (Fig. 1a, b) manifested by a decreased thickness of cardiomyocytes of the right and left ventricles (Fig. 1c, d).

A scanning electron microscopy intracellular study of cardiomyocytes showed their structural disorders following the exposure to PbO NPs, such as a loss of myofibrils and damage to the inner structure of mitochondria (Fig. 1e, f).

### Isolated Multicellular Myocardium Preparations

When studying myocardial contractility on isolated muscles (both trabeculae and papillary muscles), we established a decrease in velocity parameters (Fig. 1g) and an increase in temporal parameters (Fig. 1h, i) of a single cycle of contraction and relaxation of isolated muscles (Fig. 1). These changes were more pronounced in papillary muscles, but the same trends were observed in the trabeculae. We also noted a downward trend in isometric tension, i.e., a force of contraction normalized to the cross-sectional area of the muscle.

**Fig. 2:**
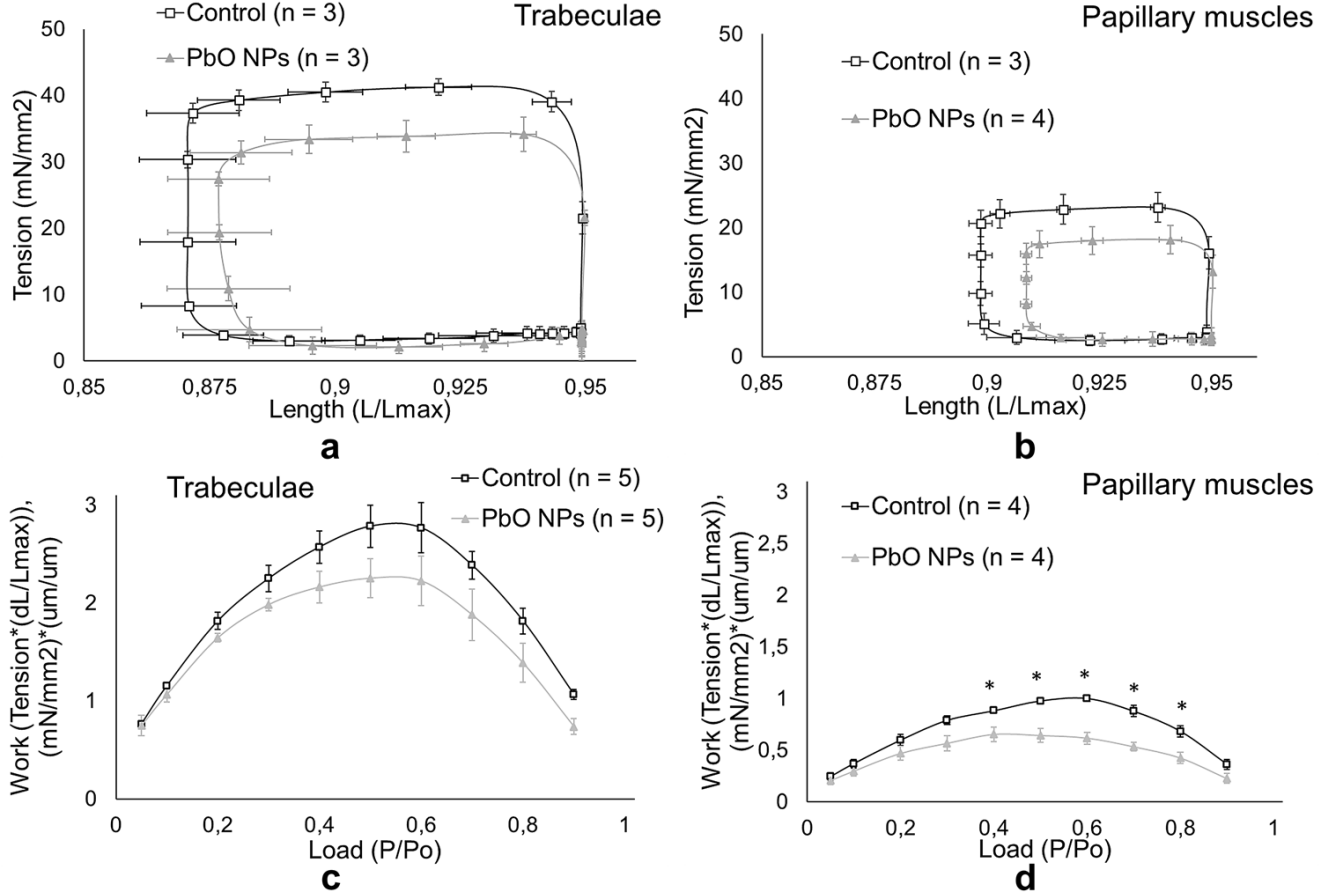
Superposition of loops “length – mechanical tension” at the afterload *P* = 0.5Po (**a**,**b**) and work performed by muscles in the afterload range of 0.05Po to 0.9Po (**c**,**d**) in the cycle of physiological contraction for trabeculae (**a**,**c**) and papillary muscles (**b**,**d**); *p < 0.05 compared with the controls

The ability of muscles to perform mechanical work is of the greatest hemodynamic importance in the whole organism (Fig. 2). The work performed by the muscles was calculated by the area of the loops, limited by the curves of changes in active mechanical stress and the length of the preparations during the contraction cycle in the physiological mode in each group of rats, followed by averaging over the entire range of afterloads. As an example, stress-length loops of trabeculae and papillary muscles are shown at an afterload of 0.5 Po (Fig. 2a, b). The decrease in the amount of work (Fig. 2c, d) performed by the muscles and calculated from the area of the loops formed by phase trajectories (Fig. 2a, b) occurs due to a decrease in the tension developed by the muscles and a decrease in the amplitude of muscle contraction. A more pronounced decrease was observed in papillary muscles (Fig. 2d).

## Discussion

### Changes in Hemodynamic Parameters

In a relatively recent review, the author noted that “In an occupational setting, the effect of lead exposure on blood pressure remains controversial” [37]. Despite that, a number of published epidemiological studies contain convincing evidence of a causal relationship between the ionic form of lead and the prevalence of arterial hypertension [13, 38–40]. Animal studies have been also carried out to identify possible mechanisms of the association between lead poisoning and hypertension [20, 21, 41–44]. We have also noted a slight increase in blood pressure following a 5-time 4-hour inhalation exposure to lead oxide nanoparticles at the concentration of 1.30 ± 0.10 mg/m^3^ in our previous experiment [45]. In this context, it should be mentioned that the air inhaled by metallurgists is contaminated with lead in the form of an aerosol containing a significant proportion of nanoparticles. Thus, our findings cannot be ignored since they indicate that even very moderate poisoning induced by exposure to PbO NPs alters blood pressure (Table 1).

A statistically significant decrease in all indicators of blood pressure (systolic, diastolic, and mean) in rats following the exposure to lead oxide nanoparticles can be due to several reasons.

First of all, a decrease in blood pressure may be associated with a weakening of blood circulation through the vessels related to insufficient oxygenation of organs and tissues, as evidenced by changes in indicators toward an increase in myoglobin in the blood serum and a decrease in hemoglobin (Table 2). It is known that hemoglobin carries oxygen to tissues, and myoglobin is involved in the storage of oxygen in it.

Secondly, hypotension can also be associated with a systemic decrease in blood flow resistance, which is consistent with a decrease in its velocity and blood filling of the tail (Table 1), as well as with the impairment of vasoconstriction processes, which can be judged by a statistically significant decrease in ACE activity and endothelin-1 concentrations (Table 2).

### Hematological Parameters and Porphyrin Metabolism

Along with myoglobinemia, the exposed rats also developed hyperenzymemia, and we established the increased activity of such enzymes as creatinine kinase, lactate dehydrogenase, aspartate aminotransferase, and gamma-glutamyltranspeptidase in the blood serum. Focus shall be made on the increase in AST activity in blood serum by 8.3 % in the exposed rodents since this very enzyme is an indicator of early damage to the heart muscle [46].

We determined the blood lead concentration as a marker of lead exposure in rats and found that it was more than 280 times higher in the exposure group compared with the controls (Table 2). An increase in the concentration of lead in the central toxicokinetic pool, i.e., in the blood, can be considered as the most important predictor of toxic effects of this metal.

With an increase in Pb in the body, the calcium content in blood serum decreased (Table 1), which demonstrates the antagonistic relationship between Pb^2+^ and Ca^2+^ [47–55].

Along with a decrease in hemoglobin during PbO NP exposure period, we observed an increase in the proportion of reticulocytes in blood and an increase in the urinary concentration of δ-aminolevulinic acid, which indicates a disorder of porphyrin metabolism. Instillation of PbO NPs in rats caused an increase in the platelet count and plateletcrit and, as a consequence, a decrease in hematocrit.

The weakening of the antioxidant defense system of the body, as evidenced by a decrease in catalase activity and the serum ceruloplasmin level, and an increased risk of atherosclerosis due to the hypoalphacholesterolemia were demonstrated.

We observed an increase in the level of intracellular enzyme creatinine kinase and its MB isoform in the blood serum, which usually occurs when the cell membrane of cardiomyocytes is damaged. Due to the hypoxia mentioned above and a potential direct damage to cardiomyocyte caused by PbO NPs [56], these intracellular enzymes enter the systemic circulation and their activity increases.

### Morphological Changes in the Myocardium

To confirm the above data on damage to the cell membrane of cardiomyocytes, we found signs of atrophy on histological sections of the myocardium (Fig. 1), which was manifested by a decrease in the thickness of cardiomyocytes of the right and left ventricles (Fig. 1).

An intracellular study of cardiomyocytes using an electron microscope revealed their structural disorders after exposure to PbO NPs, such as some loss of myofibrils and damage to the internal structure of mitochondria (Fig. 1), which negatively affected the contractile function of cardiomyocytes and, probably, bioenergetic processes inside the cell, although we did not observe changes in SDH activity in blood lymphocytes of the exposed animals (Table 1).

### Parameters of Contractility of the Heart Muscle

Identified hypoxia, calcium deficiency, and damage to the internal structures of cardiomyocytes led to impaired myocardial contractility examined on isolated muscles (trabeculae and pupillary muscles). Elongation of the contraction–relaxation cycle was shown, as assessed by a decrease in velocity parameters (Fig. 1g) and an increase in temporal parameters (Fig. 1h,i) of a single contraction–relaxation cycle of isolated muscles (Fig. 1). Those changes were more pronounced in papillary muscles, but the same trends were observed in the trabeculae. The cycle lengthening compensated for the revealed tendency to decrease in isometric tension, i.e., a force of contraction normalized to the cross-sectional area of the muscle.

A reduction in the rate of isometric contraction was probably related to changes in contractile proteins. We observed a rise in the proportion of slow-cycling V3 myosins representing a homodimer of myosin heavy chains (β-MHC 24 % against 8 % in the controls, *p*<0.05). This change at the molecular level led to a decrease in the sliding velocity of thin filaments over the bed of myosin molecules in experimental rats by 6.1 % (4.6 µm/s against 4.9 µm/s in the controls, *p*<0.05) [34].

The ability of muscles to perform mechanical work, which is of the greatest hemodynamic importance in the whole body, was reduced in both types of animal muscles following the exposure to PbO NPs, especially in papillary muscles (Fig. 2). A decrease in the amount of work (Fig. 2c,d) performed by muscles and calculated from the area of the loops formed by phase trajectories (Fig. 2a,b) occurs due to a decrease in the tension developed by the muscles and in the amplitude of muscle shortening. A decrease in the work performed by muscles can also be one of the reasons for post-exposure hypotension as it may indirectly indicate a decrease in cardiac output, which also affects blood pressure.

At the same time, the electrocardiogram showed the QT interval prolongation (Table 1), which occurs when the electrolyte balance of the body associated with hypocalcemia is disturbed [57], consistent with the data in Table 2 on the decrease in serum calcium concentrations in rats exposed to PbO NPs. In an epidemiological study, prolongation of the QT interval was found following the exposure to lead PM_2.5_ [58].

Besides, it has been noted that the QT interval prolongation is often observed in patients with anemia both at rest and during exercise [58]. Other authors found that a low hemoglobin level strongly correlated with ECG changes [58], such as ST segment depression, T wave changes, and possible QRS abnormalities.

In addition, Table 2 shows that the exposed rodents had all signs of iron deficiency anemia along with the QT interval prolongation, T wave changes, and QRS abnormalities (Table 1).

### Assumed Mechanisms of the Cardiovasotoxic Effect of Lead Oxide Nanoparticles

**Fig. 3:**
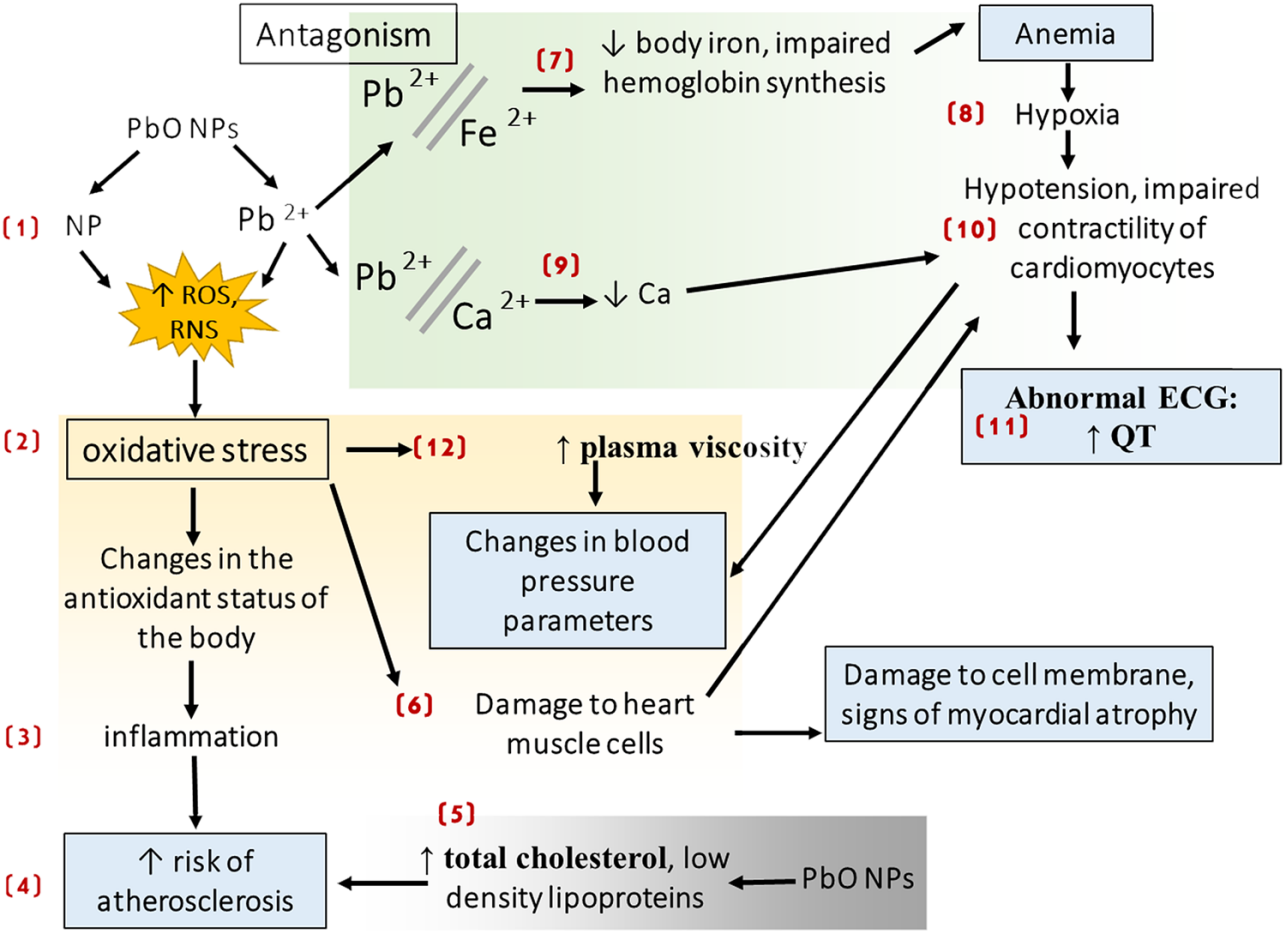
Alleged mechanisms of subchronic cardiovasotoxic effect of lead oxide nanoparticles

The assumed mechanisms of the cardiovasotoxic effect of PbO NPs following subchronic exposure are as follows (Fig. 3).

Having got into the organism, some PbO NPs dissolve and exert their effect as lead ions, while others retain their nanoscale shape (1). Their interaction with biological fluids produces reactive oxygen and nitrogen species (ROS/RNS) and leads to oxidative stress in cells (2). Oxidative stress is currently regarded as a “pathogenic partner” of the inflammatory response (3) as it activates inflammatory mediators involved in several chronic diseases [59], including atherosclerosis (4). The latter is known to be induced by accumulation of cholesterol-containing low-density lipoproteins in the intima and activation of the endothelium [60] (5), so it is, therefore, erroneous to consider lead exposure to be a trigger of atherosclerosis. Yet, it is worth considering the contribution of lead to alteration of the antioxidant status of the organism and of inflammation (3) to the development and course of the disease and the onset of possible complications (4). Besides, previous epidemiological studies have found a relationship between lead exposure and an increase in total cholesterol and low-density lipoproteins in plasma [61] (5), which is also a link in the pathogenetic mechanism of the development of atherosclerosis (4).

Excessive ROS/RNS production can induce irreversible cell damage leading to cell death as a result of necrosis and apoptosis [62], while the main targets of oxidative stress are proteins, lipids, and DNA/RNA [63]. This indicates the contribution of oxidative stress to damage to the heart muscle (6).

The abnormal heart muscle, along with anemia and a lower calcium level, contributes to impairment of myocardial contractility. Anemia that develops due to the antagonism of lead and iron (7) causes tissue hypoxia (8). Antagonistic relationships between lead and calcium (9) lead to a decrease in the level of the latter, disruption of the calcium channels, and a lower contractility of cardiomyocytes (10). We can indirectly prove these processes by lengthening of the QT interval on the ECG (11).

## Conclusions

Our experimental study of health effects of lead oxide nanoparticles (PbO NPs, mean size: 49.6±16 nm) administered during six weeks to outbred male albino rats at a total dose of 45 mg/kg body weight demonstrated toxic effects of those particles on the cardiovascular system on the cellular, tissue, organ, and organismal levels expressed by the following:

- An increase in AST activity in blood serum by 8.3% presumably indicating early damage to the heart muscle. An increase in the activity of such intracellular enzymes as creatine kinase and creatine kinase-MB in the blood serum testifies in favor of the assumption;
- Destruction of cardiomyocytes and their inner structures (a decreased thickness of cardiomyocytes and their loss of myofibrils, destruction of the mitochondrial inner structure);
- A decrease in the force of contraction of the heart muscle revealed in the evaluation of isolated muscle preparations of the myocardium of rats;
- Altered parameters of hemodynamics (lower systolic, diastolic, and mean arterial pressure) due to hypoxia and impaired vasoconstriction; and
- A higher risk of atherosclerosis, which was judged by weakening of the antioxidant system of the organism and hypoalphacholesterolemia.

The mechanisms of the cardiotoxic effect of lead oxide nanoparticles are conditioned, on the one hand, by a direct cell uptake of NPs and high oxidative stress induced by the undissolved fraction of NPs, and, on the other hand, by effects of lead ions from the dissolved particles, and include competition between lead and calcium (mimicry, replacement), Pb-induced anemia leading to disruption of tissue oxygenation and hypoxia and contributing to the increased amount of reactive oxygen and nitrogen species.

## Data Availability

The data presented in this study are available on request from the corresponding author. Mechanical and electrophysiological processes in the work of the heart differ in small rodents and large mammals. Extrapolation of data from rodents to humans shall be, therefore, done with caution.
